# Central Mechanisms of Abnormal Sympathoexcitation in Chronic Heart Failure

**DOI:** 10.1155/2012/847172

**Published:** 2012-08-07

**Authors:** Takuya Kishi, Yoshitaka Hirooka

**Affiliations:** ^1^Department of Advanced Therapeutics for Cardiovascular Diseases, Kyushu University Graduate School of Medical Sciences, 3-1-1 Maidashi, Higashi-ku, Fukuoka 812-8582, Japan; ^2^Department of Advanced Cardiovascular Regulation and Therapeutics, Kyushu University Graduate School of Medical Sciences, Fukuoka 812-8582, Japan

## Abstract

It has been recognized that the sympathetic nervous system is abnormally activated in chronic heart failure, and leads to further worsening chronic heart failure. In the treatment of chronic heart failure many clinical studies have already suggested that the inhibition of the abnormal sympathetic hyperactivity by beta blockers is beneficial. It has been classically considered that abnormal sympathetic hyperactivity in chronic heart failure is caused by the enhancement of excitatory inputs including changes in peripheral baroreceptor and chemoreceptor reflexes and chemical mediators that control sympathetic outflow. Recently, the abnormalities in the central regulation of sympathetic nerve activity mediated by brain renin angiotensin system-oxidative stress axis and/or proinflammatory cytokines have been focused. Central renin angiotensin system, proinflammatory cytokines, and the interaction between them have been determined as the target of the sympathoinhibitory treatment in experimental animal models with chronic heart failure. In conclusion, we must recognize that chronic heart failure is a syndrome with an abnormal sympathoexcitation, which is caused by the abnormalities in the central regulation of sympathetic nerve activity.

## 1. Introduction

Sympathetic nervous system has a wide variety of cardiovascular actions, including heart rate acceleration, increase in cardiac contractility, reduction of venous capacitance, and constriction of resistance vessels [1, 2]. It has already been known that abnormal autonomic nervous system regulation is involved in the pathogenesis of chronic heart failure [1–4]. Among the abnormal autonomic nervous regulation, this paper focuses on the central mechanisms of abnormal sympathoexcitation in chronic heart failure.

## 2. Sympathetic Nerve Activity Is Abnormally **** Activated in Chronic Heart Failure

Activation of sympathetic nervous system, reduction of the vagal activity, and the secretion of renin angiotensin-aldosterone axis are occurred in chronic heart failure with left ventricular systolic dysfunction [1, 2, 5] and diastolic dysfunction [6, 7]. A previous study demonstrated that the spillover of norepinephrine and epinephrine in internal jugular venous is increased in chronic heart failure [2]. Chronic heart failure is characterized by rapidly responsive arterial baroreflex regulation of muscle sympathetic nerve activity (MSNA), attenuated cardiopulmonary reflex modulation of MSNA, a cardiac sympathoexcitatory reflex related to increased cardiopulmonary filling pressure, and by individual variation in non-baroreflex-mediated sympathoexcitatory mechanisms, including coexisting sleep apnea, myocardial ischemia, obesity, and reflexes from exercising muscle [2]. In several animal models with chronic heart failure, the sensitivity of various sympathoinhibitory reflexes is reduced [8, 9]. Furthermore, experimental abnormal function of cardiovascular reflex contributes to the sympathetic activation in animal models with chronic heart failure [10]. These previous reports suggest that the reduction of sympathoinhibitory reflex is a main cause of abnormal sympathoexcitation in chronic heart failure.

 There are several animal models with chronic heart failure, and those animal models may mimic the human condition with chronic heart failure closely [11]. In spite of various methodologies, all animal models with chronic heart failure have sympathoexcitation [11], which strongly suggest that abnormal sympathoexcitation is commonly occurred in chronic heart failure, independent of its pathophysiology. In the aspect of abnormal sympathetic activation in chronic heart failure, it should be considered that abnormal central mechanisms of sympathetic nervous system regulation is occurred in chronic heart failure [3], because sympathetic nervous system activation is determined by brain [12]. Interestingly, in the patients with heart failure, significant increases in internal jugular venous spillover of metabolites of norepinephrine and epinephrine, with a positive correlation between brain norepinephrine turnover and cardiac norepinephrine spillover [2]. Moreover, central mechanisms of abnormal sympathoexcitation would be a target of the treatments for chronic heart failure.

## 3. Central Mechanisms of Abnormal ****Sympathoexcitation in Chronic Heart ****Failure: Brain Renin Angiotensin System

In the brain, renin angiotensin system is considered to be a main system of regulating sympathetic nervous system [12]. In the brain of experimental heart failure, it has been demonstrated that angiotensin II and aldosterone produced locally in the brain are related to sympathetic activation and progression of heart failure with left ventricular systolic dysfunction [9, 13]. The brain renin angiotensin system is activated in experimental chronic heart failure with enhanced central sympathetic outflow [8, 14–18]. Angiotensin II type 1 (AT_1_) receptors are found in the central nervous system and are expressed to a high degree in areas of the hypothalamus and medulla, which regulate sympathetic outflow [9, 19]. Aldosterone increases angiotensin-converting enzyme and AT_1_ receptor in the paraventricular nucleus (PVN) of the hypothalamus in chronic heart failure with postmyocardial infarction [20]. These previous reports have suggested that the activation of renin angiotensin system in the brain is associated with sympathoexcitation in chronic heart failure.

 As the mechanisms in which brain renin angiotensin system causes sympathoexcitation, brain oxidative stress has been focused. Brain renin angiotensin system is involved in the production of oxidative stress in the brain [8, 21–23]. It has been determined that mitochondria-derived oxidative stress mediates sympathoexcitation induced by angiotensin II in the brain [24, 25]. Particularly, in the brain, rostral ventrolateral medulla (RVLM) is well known as a vasomotor center [26], and oxidative stress in the RVLM causes sympathoexcitation [27]. It is well established that the AT_1_ receptor-induced oxidative stress in the RVLM causes sympathoexcitation in the animal models with chronic heart failure [8, 21, 22, 28]. Microinjection of angiotensin II into the RVLM causes sympathoexcitation, and microinjection of AT_1_ receptor blocker into the RVLM causes sympathoinhibition in experimental chronic heart failure [8, 14–18]. AT_1_ receptor protein, AT_1_ receptor mRNA, and angiotensin II levels are increased in the RVLM and nucleus tractus solitarii (NTS) in rabbits and rats with chronic heart failure [8, 21, 22]. These previous results strongly indicate that the upregulation of central AT_1_ receptor and oxidative stress plays a critical role in the abnormal sympathoexcitation in chronic heart failure. Furthermore, the balance between angiotensin-converting enzyme (ACE) and its homolog ACE2 or between AT_1_ and angiotensin II type 2 receptor in the brain may be an important determinant of sympathoexcitation in chronic heart failure [8, 25, 29, 30]. Combined these previous studies, it should be considered that the AT_1_ receptor-induced oxidative stress in the brain, especially in the RVLM, might be a novel target of the therapy for chronic heart failure through the sympathoinhibition.

## 4. Central Mechanisms of Sympathoexcitation in Chronic Heart Failure: Brain Inflammation

Brain inflammatory mediators and the brain renin angiotensin system are both implicated in sympathoexcitation in experimental chronic heart failure [31, 32]. Recently, the further central mechanisms of sympathoexcitation associated with oxidative stress are focused, such as upregulating brain proinflammatory cytokines with renin angiotensin system [33–37], perivascular macrophages in the brain [38, 39], neuronastrocyte uncoupling [40, 41], transcription factor nuclear factor kappa B (NF-*κ*B) [42], or microglial cytokines [43] in the brain. Proinflammatory cytokines, such as tumor-necrosis factor alpha, increase the number of brain perivascular macrophages, thereby activating cyclooxygenase 2 and generating prostaglandin E2, which leads to sympathoexcitation in rats with chronic heart failure after myocardia infarction [38]. There may be some interactions between proinflammatory cytokines and autonomic nervous system [44]. In addition, microglial activation with inflammation also plays an important role in sympathoexcitation [45]. Moreover, NF-*κ*B-mediates cross talk between proinflammatory cytokines and brain renin angiotensin system in rats with chronic heart failure [31, 37]. Interestingly, peroxisome proliferator-activated receptor gamma in rats with ischemia-induced heart failure is involved in the expression of inflammatory mediators and a key component of the brain renin angiotensin system in PVN, reduced sympathetic nerve activity [46]. Combined with these previous reports, brain inflammatory pathway, probably associated with renin angiotensin system, could be considered to be the important mechanisms of abnormal sympathoexcitation in chronic heart failure. Further basic and clinical experiments are necessary to determine whether the brain inflammation could be a novel target of the treatment for chronic heart failure or not.

## 5. Central Mechanisms of Abnormal ****Sympathoexcitation in Chronic Heart ****Failure: Other Possible Mechanisms

We have also demonstrated other several mechanisms of abnormal sympathoexcitation in chronic heart failure. In the brain, nitric oxide (NO) causes sympathoinhibition [47, 48], and the dysfunction of NO production in the brain occurs in the rats with chronic heart failure [49]. Overexpression of NO synthase in the brain attenuates the abnormal sympathoexcitation in mice with heart failure [50]. In the brain, NO could counteract against oxidative stress [51]. These results indicate that the dysfunction of NO pathway in the brain would cause sympathoexcitation in chronic heart failure. Moreover, it has been demonstrated that each of small G protein Rho/Rho kinase pathway, mineral corticoid receptors and/or Na sensitivity, or toll-like receptor 4 in the brain causes sympathoexcitation in rats with chronic heart failure [52–55]. It would be necessary to clarify whether these various mechanisms have interaction with brain renin angiotensin system and/or inflammation in chronic heart failure with sympathoexcitation.

## 6. Sympathoinhibitory Therapy for **** Chronic Heart Failure

Many clinical studies have already and strongly suggested that chronic beta blocker therapy improves left ventricular performance and reverses left ventricular remodeling, reduces risk of hospitalization for heart failure, and improves survival of chronic heart failure [56–61]. Among all beta blockers, bisoprolol (except in the USA), carvedilol, and metoprolol succinate (except in Canada) are almost universally approved for the treatment of chronic heart failure [56–61]. However, previous studies could not demonstrate the benefits of alpha1-blocker in chronic heart failure [62–64].

Central alpha2 receptor has been considered to be possible targets of treatment for chronic heart failure, because the excitation of the central alpha2 receptor causes sympathoinhibition [9, 65]. In modest doses of clonidine, it significantly attenuates cardiac and renal sympathetic tone in the patients with chronic heart failure [66]. The other centrally acting sympathoinhibitory agent, moxonidine, acts through both alpha2- and imidazoline receptors [9, 67]. However, in clinical trials, moxonidine led to increased mortality [9, 68].

 Angiotensin II and aldosterone production enhances the release and inhibits the uptake of norepinephrine at nerve endings [69]. ACE inhibitors have a predictable effect in increasing plasma renin and decreasing angiotensin II and aldosterone levels, whereas norepinephrine and vasopressin reduction is attributed to the hemodynamic improvement [70]. Previous large clinical trial has already shown the benefit with aldosterone antagonists in patients with chronic heart failure and may be partially related to their effect on norepinephrine [71]. The high density of AT_1_ receptors is present in brain regions outside of the blood-brain barrier where peripherally administered AT_1_ receptor blockers are able to access without considering the existence of the blood-brain barrier as well as inside of the blood-brain barrier [72]. Recent studies suggest that the systemic administered AT_1_ receptor blockers also act on the AT_1_ receptors within the brain, thereby reducing blood pressure in hypertensive rats [51, 73–77]. It should be determined in future studies whether ACE inhibitors or AT_1_ receptor blockers could cause beneficial sympathoinhibition via blockade of brain renin angiotensin system.

 Several studies in rabbits with pacing-induced heart failure have demonstrated that statins normalize abnormal sympathetic hyperactivity in experimental chronic heart failure [78–80]. Previous studies have suggested that simvastatin could inhibit AT_1_ receptor and production of superoxide with upregulating NO synthase in the RVLM of the animal models with chronic heart failure [78, 80]. We also demonstrated that orally administered atorvastatin causes sympathoinhibition and improves baroreflex dysfunction via reduction of oxidative stress and upregulation of NO synthase in the brain of hypertensive rats [81–83]. Although there is no clinical study suggesting the benefits of statins on chronic heart failure, these experimental results suggest that stains could attenuate sympathoexcitation in chronic heart failure, independent of cholesterol-lowering effect. Further clinical trials are necessary to clarify whether statins in clinical dose would have the sympathoinhibitory benefit in chronic heart failure or not.

As the nonpharmacological therapy for chronic heart failure, exercise training is considered to have sympathoinhibitory benefit on chronic heart failure. Exercise intolerance is a characteristic of patients with chronic heart failure, and skeletal myopathy contributes to the limitation of functional capacity in chronic heart failure [1, 2, 9]. Abnormal sympathetic hyperactivity contributes to the skeletal myopathy in chronic heart failure [84]. Interestingly, current evidences have suggested that exercise training improves central hemodynamics, peripheral muscle function, and symptoms and causes sympathoinhibition even in patients treated with beta blockers [85–88]. Recent experimental evidence suggests that the exercise training-induced beneficial effects on autonomic activity in heart failure may be due to an upregulation in central antioxidative mechanisms and suppressed central pro-oxidant mechanisms [29].

 Recent novel topic in the therapy with sympathoinhibition is renal sympathetic denervation. Renal afferent nerves may also contribute to the blood pressure elevation according to the recent findings of the renal nerve ablation in patients with resistant hypertension [89–91]. Renal afferent nerves project directly into many areas in the central nervous system controlling the sympathetic nervous system activity [92–94]. We consider that the renal nerve ablation could be a novel therapy for chronic heart failure, and further clinical trials and basic researches are expected.

## 7. Summary and Future Prospects

In chronic heart failure, it has been recognized that the abnormal sympathoexcitation occurs. In the treatment of chronic heart failure, the therapy with sympathoinhibition, such as beta blockers and/or exercise training, has already been considered to be important. We must recognize that chronic heart failure is a complex syndrome with sympathoexcitation and that the abnormal sympathoexcitation should be the target of the treatments for chronic heart failure. In this aspect, conservative pharmacological therapy is not sufficient, and the additive new and novel device therapy and/or nonpharmacological therapy are necessary.

**Figure 1:**
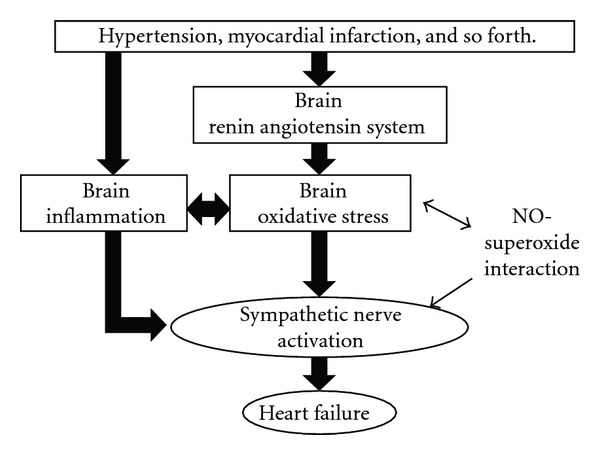
A schema of the concept in the central abnormalities of regulation for sympathetic nerve activity in chronic heart failure.

 The mechanisms in which the abnormal sympathoexcitation occurred in chronic heart failure have not been fully determined. Particularly, the central abnormalities need further examinations in clinical and basic research. It is interesting and important to consider that AT_1_ receptors, oxidative stress, and inflammatory pathway in the brain are novel sympathoinhibitory therapeutic targets for chronic heart failure (Figure 1).
